# A randomized controlled trial of hospital versus home based therapy with oral amoxicillin for severe pneumonia in children aged 3 – 59 months: The IndiaCLEN Severe Pneumonia Oral Therapy (ISPOT) Study

**DOI:** 10.1186/s12887-015-0510-9

**Published:** 2015-11-17

**Authors:** Archana B. Patel, Akash Bang, Meenu Singh, Leena Dhande, Luke Ravi Chelliah, Ashraf Malik, Sandhya Khadse, ISPOT Study Group

**Affiliations:** Lata Medical Research Foundation and Indira Gandhi Government Medical College, Nagpur, India; Mahatma Gandhi Institute of Medical Sciences, Sewagram, Maharashtra India; Post Graduate Institute of Medical Sciences, Chandigarh, India; Institute of Child Health, Chennai, India; Jawaharlal Nehru Medical College, Aligarh Muslim University, Aligarh, India; B.J. Medical College, Pune, India

**Keywords:** Severe pneumonia, Lower chest indrawing, Hospitalization, Oral amoxicillin, Cost effective, Randomized trial

## Abstract

**Background:**

Pneumonia is the leading cause of child mortality under five years of age worldwide. For pneumonia with chest indrawing in children aged 3–59 months, injectable penicillin and hospitalization was the recommended treatment. This increased the health care cost and exposure to nosocomial infections. We compared the clinical and cost outcomes of a seven day treatment with oral amoxicillin with the first 48 h of treatment given in the hospital (hospital group) or at home (home group).

**Methods:**

We conducted an open-label, multi-center, two-arm randomized clinical trial at six tertiary hospitals in India. Children aged 3 to 59 months with chest indrawing pneumonia were randomized to home or hospital group. Clinical outcomes, treatment adherence, and patient safety were monitored through home visits on day 3, 5, 8, and 14 with an additional visit for the home group at 24 h. Clinical outcomes included treatment failure rates up to 7 days (primary outcome) and between 8–14 days (secondary outcome) using the intention to treat and per protocol analyses. Cost outcomes included direct medical, direct non-medical and indirect costs for a random 17 % subsample using the micro-costing technique.

**Results:**

1118 children were enrolled and randomized to home (*n* = 554) or hospital group (*n* = 564). Both groups had similar baseline characteristics. Overall treatment failure rate was 11.5 % (per protocol analysis). The hospital group was significantly more likely to fail treatment than the home group in the intention to treat analysis. Predictors with increased risk of treatment failure at any time were age 3–11 months, receiving antibiotics within 48 h prior to enrolment and use of high polluting fuel. Death rates at 7 or 14 days did not differ significantly. (Difference −0.0 %; 95 % CI −0.5 to 0.5). The median total treatment cost was Rs. 399 for the home group versus Rs. 602 for the hospital group (*p* < 0.001), for the same effect of 5 % failure rate at the end of 7 days of treatment in the random subsample.

**Conclusions:**

Home based oral amoxicillin treatment was equivalent to hospital treatment for first 48 h in selected children of chest indrawing pneumonia and was cheaper. Consistent with the recent WHO simplified guidelines, management with home based oral amoxicillin for select children with only fast breathing and chest-indrawing can be a cost effective intervention.

**Trial Registration:**

ClinicalTrials.gov NCT01386840, registered 25^th^ June 2011 and the Indian Council of Medical Research REFCTRI/2010/000629.

**Electronic supplementary material:**

The online version of this article (doi:10.1186/s12887-015-0510-9) contains supplementary material, which is available to authorized users.

## Background

Pneumonia is the single largest killer of children under the age of five worldwide [1]. The disease kills over two million children under the age of five every year— nearly one fourth (400,000) of these deaths occur in India alone [2]. About half of pneumonia cases in India are caused by bacteria and could be treated with antibiotics. However, only 13 % of Indian children under the age of five with suspected pneumonia receive antibiotics [3].

The 2008 WHO guidelines for treatment of non-severe pneumonia (cough, fever and fast breathing) recommend health workers to provide oral antibiotics for three days at home but urgent referral for hospitalization for parenteral (injectable) antibiotics and other supportive therapy after administration of first dose of antibiotics, if the child has severe pneumonia (cough, fever, fast breathing and lower chest indrawing) or very severe disease (pneumonia with the presence of WHO defined danger signs) [4]. Often inability to access a referral facility deprives these children from getting appropriate care. For many families, seeking treatment for their children at a health care facility is often logistically and financially burdensome thus denying them early administration of antibiotics within 48 h that can potentially improve their outcomes. Additionally transport to a distant facility can entail serious delays in effective treatment. Many children with severe pneumonia referred for admission to a hospital could die in transit or reach too sick to be saved [5]. In addition, when hospitalized, the children with severe pneumonia are vulnerable to nosocomial infections in crowded hospital wards and are also at risk of needle-borne infections due to parenteral therapy. Two important studies have addressed such barriers to the recommended treatment of severe pneumonia. The first study was intended to determine whether oral antibiotics are equivalent to injectable antibiotics when both are given in the hospital. This was an open label equivalency study called APPIS (Amoxicillin Penicillin Pneumonia International Study), which was a large multicentre randomized controlled trial comparing injectable penicillin versus oral amoxicillin given for 7 days to children in the hospital [6]. The second study was called “NO-SHOTS” (New Outpatient Short-Course Home Oral Therapy for Severe Pneumonia Study) and was a randomized, open-label equivalency trial done at seven study sites in Pakistan and compared initial hospitalization and parenteral ampicillin for 48 h followed by 3 days of oral amoxicillin at home, to 5 days of home-based treatment with oral amoxicillin [7]. NO-SHOTS showed that home treatment with high-dose oral amoxicillin is equivalent to hospital based treatment with parenteral ampicillin in selected children aged 3–59 months with WHO defined severe pneumonia [7]. Later, another study- the MASS study (Multicenter Amoxicillin Severe pneumonia Study) showed that clinical treatment failure and adverse event rates among children with severe pneumonia treated at home with oral amoxicillin did not substantially differ across geographic areas (Bangladesh, Ghana, Vietnam and Egypt) and hence home-based therapy of severe pneumonia could possibly be applied to a wide variety of settings [8]. Thus oral amoxicillin at home has proven clinically efficacious in various settings across the world for treatment of selected children with WHO defined severe pneumonia. The Lancet Series on Childhood Pneumonia and Diarrhoea has reported that case management is one of the three most effective interventions to reduce pneumonia deaths in children but also noted that the cost effectiveness of these interventions in national health systems needs urgent assessment [9]. So the cost savings or cost-effectiveness of home-based oral antibiotic treatment for WHO defined severe pneumonia in childhood would be important to inform public policy and has not been previously evaluated.

Therefore our objective was to assess the efficacy and cost-effectiveness of a 7-day home-based course of oral amoxicillin as compared to oral amoxicillin administered for the first 48 h in the hospital followed by 5 days of home-administration.

## Methods

We conducted an open labelled multi-center prospective two-arm randomized clinical trial at 6 referral hospitals in India (Chandigarh, Chennai, Nagpur, Pune, Sewagram and Aligarh) to evaluate the difference of rates of treatment failures of a 7-day course of oral amoxicillin when administered at home as compared to a 7-day course of oral amoxicillin administered for the first 48 h in the hospital followed by 5 days of home-administration to treat WHO defined severe pneumonia in children aged 3–59 months. In addition to the clinical outcomes, the costs of treating severe pneumonia, the differences in costs of treatment in the two study groups and the cost-effectiveness of the two alternative treatment strategy was also assessed in this trial.

The study was approved by the institutional ethics committees of: Indira Gandhi Government Medical College, Nagpur; Post Graduate Institute of Medical Sciences, Chandigarh; Government General Hospital, Chennai; B.J. Medical College, Pune; Mahatma Gandhi Institute of Medical Sciences, Sevagram, Wardha; Jawaharlal Nehru Medical College, Aligarh; the Research Ethics Review Committee, World Health Organization; and the INCLEN Institutional Review Board through the India Clinical Epidemiology Network (IndiaCLEN, dated 18^th^ Nov 2006).

### Eligibility

Children aged 3–59 months with cough/difficulty in breathing of less than 2 weeks duration, lower chest indrawing (LCI), unresponsive to nebulisation, who did not have any of the exclusion criteria (Table 1) and whose parents gave a written informed consent for their participation were enrolled in the study by trained research staff. All included children were administered the first dose of amoxicillin, sent for chest radiology and then reassessed after radiology. Children were randomized to either treatment arms if there was no clinical deterioration or radiographic signs of consolidation, effusion or pneumothorax (using the the WHO manual for standardization of interpretation of chest radiographs for the diagnosis of pneumonia in children) [10].

**Table 1:** Exclusion Criteria

1. Known or clinically recognizable chronic conditions
2. History of > 2 weeks of cough /difficulty in breathing
3. Past history of more than 3 wheezing episodes or physician diagnosed asthma
4. LCI that responds to trial of nebulization
5. Respiratory rate (RR) >70 breaths per minute in calm child
6. Known HIV positive child or HIV status of mother known to be positive and status of child not known/defined.
7. Hospitalization for > 48 h in the last two weeks
8. Measles in the last month
9. Clinically severe malnutrition (weight for length < −3 SD or kwashiorkor) (refer to WHO growth chart)
10. Rickets
11. Central cyanosis
12. Kerosene poisoning within last 48 h
13. Oxygen saturation (pulse oximetry) <88 % on room air
14. Abnormally sleepy or difficult to wake
15. Inability to drink
16. Stridor in calm child
17. Convulsions during this illness
18. Known any antibiotic therapy for 48 h or more immediately prior to admission
19. Other diseases requiring antibiotic therapy, e.g. Meningitis, tuberculosis, dysentery, etc.
20. Persistent vomiting (>3 episodes of vomiting within 1 h)
21. Grunting
22. Known prior anaphylactic reaction to penicillin or amoxycillin
23. Severe dehydration according to WHO guidelines
24. Severe pallor
25. Suspected surgical pathology
26. Living out of the follow-up area of the study (30 kms)
27. Subject previously included in the same trial or already included in another ongoing trial anywhere
28. Presence of radiological consolidation / effusion / pneumothorax

### Randomization

Random numbers were computer generated, by using variable length permuted blocks at the coordinating site using STATA 10 program. A separate list was generated for each site and the individual patient assignments were placed in a series of sealed opaque envelopes that were opened for serially eligible patients. The eligible study participants were randomly allocated to either the hospital group in which syrup amoxicillin (50 mg/kg/d in two divided doses) was administered in hospital for initial two days by hospital staff followed by administration at home for five days by the care-giver, or, to the home group in which the first dose of amoxicillin was administered in hospital and subsequent doses were administered by the care-giver at home for seven days.

### Data collection

Clinical and demographic data was collected at baseline along with throat swab and nasopharyngeal aspirate. Both groups were followed up through home visits on day 3, 5, 8 & 14 and home group had an additional home visit at 24 h. During these follow-up home visits, data regarding outcomes were collected. This included clinical deterioration of disease any time after enrolment, change of antibiotics, hospitalization, serious adverse events considered related to amoxicillin, left against medical advice (LAMA), voluntary withdrawal of consent, or loss to follow up.

### Clinical outcomes

Treatment failure was defined as presence of any one of the following conditions - clinical deterioration of disease any time after enrolment that required change of antibiotics, hospitalization (any time for the children in the home managed group or clinical decision to extend the hospitalization longer than 48 h in the hospitalized group), an occurrence of a serious adverse event related to amoxicillin, left against medical advice (LAMA), voluntary withdrawal of consent from the study, or loss to follow up. Clinical deterioration was defined as appearance of signs of very severe disease such as persistent vomiting (vomiting repeated three times within an hour due to any reason), central cyanosis, grunt, stridor, abnormal sleepiness or difficulty to wake, inability to drink, SpO2 < 85 %, convulsions, or death [6]. Antibiotics would be changed if there was clinical deterioration, developing a co-morbid condition, or, persisting fever > 98.6 °F with lower chest indrawing even after 3^rd^ day, or, fever alone at day 5, or, lower chest indrawing alone (non responsive to three doses of nebulisation with bronchodilator) at day 5 (as reported by the mother), or, persistence of fast breathing after day 7 which is unresponsive to three doses of nebulization with bronchodilator. Rigorous training and retraining of the research physicians using standard operating procedures was used to minimize the biases that may arise due to lack of uniformity in assessing clinical signs between treatment groups and across sites. Strong quality monitoring processes were also established. An additional file describes the relevant standard operating procedures in details [see Additional file 1].

### Cost outcomes

Cost data were collected for 17.2 % patients starting from before enrolment till day 14 or till the patient recovered whichever was earlier. Three distinct types of forms were used at enrolment, daily in the hospital and at each visit respectively. The cost data were also collected for those patients who left against medical advice. The forms included information about the service provider and the type of service. We disregarded fixed costs that were common for the two strategies. The protocol driven costs, such as investigations required for the study but not otherwise conducted routinely, were excluded from calculation of these costs. The variable costs i.e. direct medical, direct non-medical and indirect costs of the two treatment arms were measured using micro-costing technique [11].

**Direct medical costs** included costs of medical resources utilized by the patient at the out-patient and during hospital stay as calculated from the patient's perspective eg cost of medications, physician and nurses services and other paramedical services, bed cost, and the laboratory investigations.

**Direct non-medical costs** included the cost of travelling to the hospital for the patient and the family, cost of food to the family and patient during hospitalization and other incidental cost to the family attributed to the illness.

**Indirect costs** were measured by the lost wages for employed parents or guardians attending to the participant.

The median differences in costs and the predictors of total cost were analyzed as cost data was not normally distributed. The incremental cost-effectiveness of the two treatment strategies was also assessed.

### Sample size

Sample size estimates were based to detect equivalence and on the hypothesis that children who were treated with oral amoxicillin at home would experience a failure rate of 15 %, and, would be within 5 % of those treated for first 48 h in hospital. The estimated sample size was 1,234 i.e. 617 per group. The sample size was calculated for the clinical trial but provided 90 % power for a two-tailed alternative hypothesis to calculate a mean difference in costs between the two interventions.

### Statistical analysis

Baseline characteristics of the two treatment groups were compared using chi-square tests for categorical variables and ANOVA for continuous variables. We conducted the analysis using both intention to treat (analyzed as randomized) and per protocol analysis (included all clinical causes of treatment failure, but excluded treatment failure due to lost to follow up, LAMA, and voluntary withdrawal from the study). Cox proportional hazards models were used to estimate the relative hazards (RH) of treatment failure in the two groups up to 14 days and to explore associations between the same baseline explanatory covariates (age, feeding status, immunization, antibiotics prior to 48 h, weight for age Z scores, body temperature, respiratory rates, oxygen saturation, auscultatory wheeze, crackles, radiological infiltrates, number of rooms in the house and type of fuel used for cooking) and outcome. We used forward step wise method and identified explanatory candidate variables (*p* ≤ 0.1) for inclusion in adjusted models as plausible predictors of treatment failure. The Kaplan-Meier curves for the cumulative probability of treatment success were also plotted for the two groups and the overall difference in their rates of treatment success was examined using the log-rank test. Statistical analysis was conducted using STATA 10 data analysis software.

### Economic analysis

The medians of the direct medical, direct non-medical and indirect costs and their inter-quartile ranges were calculated. Group differences in median costs of the treatment strategies were assessed using the median test. Univariate analysis was conducted for the predictors of cost variation, such as data on patient demographic characteristics, clinical history, length of stay and other utilization of resources for treatment of this episode of pneumonia before entry of patients into the trial. Multivariable regression analysis (OLS, with log transformation) was also used to predict total costs across the cost categories using pre-randomization variables, the alternate treatment strategies and other covariates that relate to resource consumption. Differences were considered statistically significant if they had a two-tailed* p* value less than 0.05. The hypothesis, that home treatment is more cost-effective than hospital treatment, was also tested by comparing the cost-effectiveness ratios. The incremental cost-effectiveness was estimated as the difference in the predicted total costs in the numerator and the difference in effects i.e. the number of patients cured (1-treatment failure) or the number of cases of treatment failure avoided in the denominator.

## Results

**Fig. 1:**
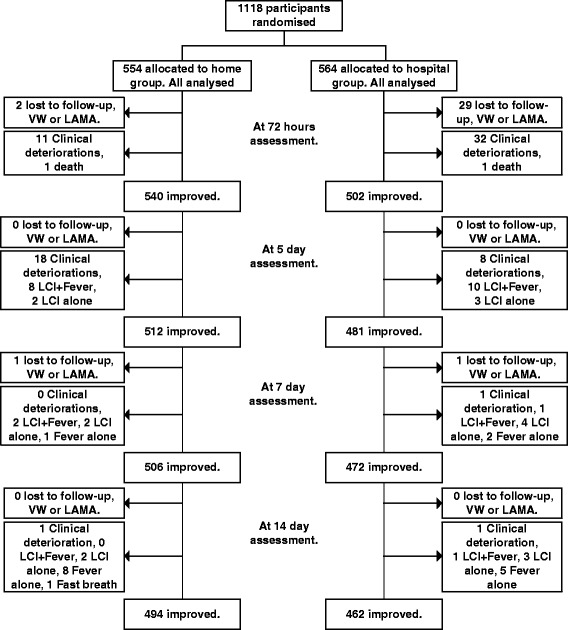
Trial profile

The study was conducted from October 2008 to March 2011. Of the children screened for WHO defined severe pneumonia, 1118 (16.9 %) were enrolled, 554 were assigned to home treatment, and 564 were assigned to hospital treatment, across six sites in India (Fig. 1). The number of children enrolled from different sites were 377 (33.7 %), 328 (29.3 %), 316 (28.3 %), 50 (4.5 %), 37 (3.3 %), and 10 (0.9 %) from Chandigarh, Chennai, Nagpur, Sewagram, Aligarh, and Pune respectively. The reasons for excluding 83.1 % of screened children are shown in Table 2. The two intervention groups were not statistically different in their baseline characteristics (Table 3).

**Table 2:** Study Screening and Reasons for Exclusion

	Total^#^	
	n	%^a^
Screened	6,634	
Enrolled (%)	1,118	16.9
Inclusion criteria not satisfied		
Not satisfying Age 3–59 months	462	5.1
Had no lower chest indrawing on examination	1489	16.3
Caretaker is not willing to sign informed consent form	4060	44.5
Exclusion criteria, number (%)		
Known or clinically recognizable chronic conditions	224	2.5
History of >2 weeks of cough / difficulty in breathing	195	2.1
Past history of more than 3 wheezing episodes or physician diagnosed asthma	267	2.9
LCI that responds to trial of nebulization	743	8.1
Known HIV positive child. HIV status of mother known to be positive & of child not known/defined	16	0.2
Hospitalization for > 48 h in the last two weeks	127	1.4
Measles in the last month	64	0.7
Clinically severe malnutrition (weight for length < −3 SD or kwashiorkar)	163	1.8
Rickets	21	0.2
Kerosene poisoning within last 48 h.	17	0.2
Antibiotic therapy for 48 h or more immediately prior to admission	431	4.7
Other diseases requiring antibiotic therapy, e.g. Meningitis, tuberculosis, dysentery, etc.	28	0.3
Known prior anaphylactic reaction to penicillin or amoxycillin	8	0.1
Severe dehydration according to WHO guidelines	12	0.1
Severe pallor	19	0.2
Suspected surgical pathology	12	0.1
Living out of the follow-up area of the study (30 kms)	308	3.4
Subject previously included in the same trial or already included in another ongoing trials anywhere.	44	0.5
Presence of danger sign ^c^ before radiology evaluation	358	3.9
Presence of danger sign after radiology evaluation	9	0.1
Presence of radiological consolidation/ effusion / pneumothorax	45	0.5
Total reasons for exclusion^b^	9122	

^a^Proportion for the presence of exclusion criteria. The denominator is total reasons for exclusion2s

^b^A child could have more than one reason for exclusions

^c^Danger signs are presence of any one clinical condition - abnormally sleepy or difficult to wake, persistent vomiting, inability to drink, grunting, stridor, central cyanosis, convulsions, RR > 70 breaths per minute, Sp02 < 88 % on room air

**Table 3:** Baseline Characteristics in Home and Hospitalized Children

Baseline characteristic	Home (*N* = 554)		Hospital (*N* = 564)	
	*n*	%	*n*	%
Sex (Female)	207	37.4	204	36.2
Infants (3–11 months old)	237	42.8	277	49.1
Children (12–23 months old)	164	29.6	138	24.5
Children (24–59 months old)	153	27.6	149	26.4
Breast feeding indicators(3-59months)				
Exclusive breast feeding	361	65.2	373	66.1
Bottle feeding	231	41.7	209	37.1
Timely complementary feeding	431	77.8	443	78.6
Immunization status up-to-date	516	93.1	510	90.4
Report of antibiotics in < 48 h prior to enrollment	56	10.1	59	10.5
Weight-for-age Z score (mean ± sd)	554	−1.5 ± 1.3	564	−1.6 ± 1.3
Weight-for-age Z score (<−2)	197	35.6	205	36.4
Length/ height (cm)(mean ± sd)	548	75.9 ± 11.7	560	74.7 ± 12.1
Temperature (°F)(mean ± sd)	554	99.5 ± 1.5	564	99.6 ± 1.5
Respiratory rate per min (mean ± sd)				
Infants	237	47.3 ± 8.9	277	48.9 ± 8.7
Children	317	43.0 ± 9.1	287	43.9 ± 9.8
Auscultatory wheeze				
Infants	140/237	59.1	176/277	63.5
Children	155/317	48.9	121/287	42.2
Crackles				
Infants	174/237	73.4	211/277	76.2
Children	243/317	76.7	228/287	79.4
Pulse oximetry (mean ± sd)				
Infants	236	96.1 ± 2.9	277	95.9 ± 3.2
Children	317	95.9 ± 3.0	286	95.8 ± 3.1
Any Infiltrates in chest x-ray	322/530	60.8	365/540	67.6
No. of rooms in house (mean ± sd)	554	2.4 ± 1.4	564	2.3 ± 1.4
Fuel used for cooking				
Low polluting fuel	220/553	39.8	249/556	44.8
High polluting fuel	333/553	60.2	307/556	55.2
Any smoker who smokes in the house	121	21.8	129	22.9

### Clinical outcomes

The cumulative overall treatment failure (home + hospital) on oral amoxicillin at different time points were 6.8 % at <72 h, 11.2 % at <5 days, 12.5 % at <7 days, and 14.5 % at <14 days by intention to treat and 4 % at <72 h, 8.4 % at <5 days, 9.6 % at <7 days, and 11.5 % at <14 days respectively by per protocol analysis.

**Fig. 2:**
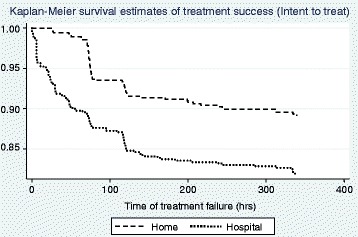
Kaplan Meier Curves for treatment success rates for intention to treat analysis

The treatment failure rate at 14 days in hospital group was 18.1 % (102/564) as compared to 10.8 % (60/554) in the home group. There were 30 (5.4 %) failures due to clinical deterioration (presence of any one of these conditions - persistent vomiting, central cyanosis, grunt, stridor, abnormally sleepy or difficult to wake, inability to drink, or convulsions) in the home group and 42 (7.4 %) in the hospital group (Fig. 1) which was not significantly different. The failures due to LAMA or voluntary withdrawal were significantly more in hospital group as compared the home group. [5.3 % (30/564) vs 0.5 % (3/554); *p* < 0.001] Kaplan Meier curves for differences between treatment successes in the home and hospital group with log rank tests for the intention to treat (ITT) analysis showed that the hospital group was significantly more likely than home children to fail treatment at any time point. (HR 1.79; 95 % C.I. 1.30, 2.46, p < 0.01) (Fig. 2) The per protocol analysis, though tended to show a similar trend (RR 1.32), was statistically non-significant (*p* = 0.10). The Cox Regression model showed that infants (3–11 months) and patients who had antibiotics within 48 h of enrolment had a higher likelihood of failing treatment at any point from enrolment to 14 days (per protocol and intention to treat analysis). Additionally, belonging to the hospital group and residence in homes with high polluting fuels were significantly associated with treatment failure in ITT, because children in the hospital group were more likely to fail treatment at any time than children in the home group (with LAMA accounting for the majority of these failures) (Table 4).

**Table 4:** Cox Regression Analysis for Treatment Failure Using the Per Protocol and Intention to Treat Analysis up to 14 days

Characteristic	Per-protocol analysis				Intention to treat analysis			
	Hazard ratio	95 % CI		*p*-value	Hazard ratio	95 % CI		*p*-value
Treatment group								
Home	1.00				1.00			
Hospital	1.32	0.93	1.88	0.12	1.61	1.16	2.24	0.00
Age group								
Infants (3–11 months old)	1.00				1.00			
Children (12–59 months old)	0.65	0.45	0.93	0.02	0.69	0.49	0.96	0.03
Exclusive breast feeding								
No	1.00				1.00			
Yes	1.52	0.98	2.36	0.06	1.46	0.99	2.16	0.06
Antibiotics prior to enrollment								
No	1.00				1.00			
Yes	1.79	1.06	3.02	0.03	1.72	1.07	2.76	0.02
Fuel used for cooking								
Low polluting fuel	1.00				1.00			
High polluting fuel	1.43	0.98	2.10	0.06	1.51	1.06	2.15	0.02
Study Site								
Chandigarh	1.00				1.00			
Chennai	0.18	0.09	0.33	0.00	0.41	0.26	0.64	0.00
Nagpur	0.58	0.38	0.91	0.02	0.63	0.42	0.96	0.03
Pune	2.33	0.84	6.48	0.11	2.78	1.11	6.97	0.03

Two children died within the first 72 h, one in the home group, and the other in the hospital group. There were no other serious adverse events. Neither of the deaths were considered to be related to the study treatment with oral amoxicillin.

### Cost outcomes

**Fig. 3:**
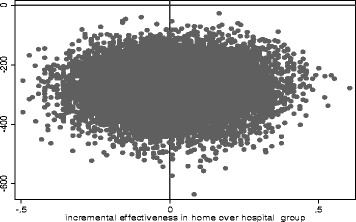
20000 Bootstrap re-samples –cost-effectiveness plane

The average cost of treating a child at a government hospital in India with subsidized rates was Rs. 567 when patients were hospitalized for only two days. The median total cost for treating at home was significantly less than treating at hospital for the first 48 h. (Rs 399 for home vs. Rs 602 for the hospital group, *p* < 0.001) (Table 5) The predictors of total mean costs of treatment are shown in Table 6. The patient characteristics associated with higher costs of treatment were age 3–11 months, higher temperature, lower pulse oximetry readings, and presence of auscultatory wheeze. The boot strap cost estimates of Rs.702 (95 % CI 701, 703) for the hospital group and Rs. 427 (95 % CI 427, 428) for the home group were consistent with those determined by the above regression and were significantly higher for the hospital group (*p* < 0.001). Figure 3 shows the plotting of the boot strap estimates (20,000 re-samples) on the cost effectiveness plane. It indicates that it is cheaper to be treated at home (all points are below zero in the Y co-ordinate of costs) with identical effects (all points are equally distributed on either side of zero in the X co-ordinate of effectiveness).

**Table 5:** Treatment costs in different cost categories in the home and hospital group

Type of costs	Home		Hospital		*P*-value*
	*n*	(median ± IQR)	*n*	(median ± IQR)	
Cost of outpatient visits					
Direct Medical Cost	94	104 ± 213	97	83 ± 215	0.6
Direct Non Medical cost	94	57.5 ± 68	97	60 ± 85	0.7
Indirect Cost	86	0 ± 0	84	0 ± 0	0.5
Hospital Cost					
Direct Medical Cost	76	130 ± 36	95	166 ± 101	<0.001
Direct Non Medical cost	76	40 ± 37	95	165 ± 130	<0.001
Indirect Cost	76	0 ± 50	95	0 ± 150	0.02
Total Direct Medical Cost	94	208 ± 204	97	215 ± 236	0.09
Total Direct Non Medical cost	94	90 ± 91	97	225 ± 210	<0.001
Total Indirect Cost	94	0 ± 100	97	0 ± 150	0.1
Total Cost	94	399 ± 346	97	602 ± 524	<0.001

*P-value of median test

**Table 6:** Predictors of total mean costs for treating WHO defined severe pneumonia

Variable	β (95 % CI)	*P*-value
Treatment group (Hospital)	239.8 (102.6, 377.0)	0.00
Age group (12–59 months old)	−195.7 (−341.6, −49.9)	0.01
Antibiotics prior to enrollment	−22.7 (−297.5, 252.1)	0.87
Weight for age Z-score	−36.4 (−94.0, 21.2)	0.21
Temperature	28.1 (3.9, 52.2)	0.02
Respiratory rate per min	1.9 (−6.1, 10.0)	0.64
Pulse oximetry	−26.9 (−50.4, −3.4)	0.03
Auscultatory wheeze	204.8 (59.5, 350.1)	0.01
Any infiltrates in chest X-ray	106.8 (−37.7, 251.3)	0.15

## Discussion

### Is oral Amoxicillin effective?

Our study showed that oral amoxicillin, whether administered at hospital or at home for the first 48 h was effective in treating WHO defined severe pneumonia in 93.2 % of eligible patients who were otherwise clinically stable and did not have co-morbid conditions. Although the rates of clinical deterioration were similar over the 14 day follow up, the treatment failure rate was more in the hospital group (18.1 % vs 10.8 %), due to higher rates of LAMA/voluntary withdrawal (5.3 % in hospital group vs 0.5 % in home group), one of the criteria for the composite outcome “treatment failure”. This was a conservative estimate of treatment success as there is uncertainty of the clinical outcome and treatment adherence in those who leave the study prematurely. Figure 1 describes frequency of presence of various criteria for treatment failure and of true clinical deterioration. Secondly, children who are hospitalized are closely monitored by skilled research staff for presence of signs of clinical deterioration and are also likely to experience a change in antibiotic (also a criterion for treatment failure) by a treating physician. These could have potentially increased the failure rate in the hospital group. However, clinical deterioration at < 7 days was not significantly different between the groups indicating that it did not cause a potential bias in this study. Selective randomization of sicker children to the hospital was unlikely as the allocation was concealed. The high rate of LAMA in the hospital group (5.1 %) demonstrates that hospitalization is a barrier to children receiving a full course of treatment and perhaps the caregivers prefer to have their children who otherwise do not have co-morbid conditions such as those listed out in the Table 1, treated at home.

The baseline characteristics predictive of treatment failure were known risk factors such as younger age 3–11 months [12, 13], those who received antibiotics less than 48 h prior to enrollment (perhaps clinically sicker children) and use of solid fuels for household cooking, a known risk factor for poor treatment response [14–18]. Also, all other sites had lower failure rates than Chandigarh. This was mostly because of persistence of LCI at 48 h contributed mostly by children of hyperreactive airway disease. National Family Health Survey 2005–06 has reported the northern states to have higher prevalence and symptoms of acute respiratory infections [3]. Chandigarh is in the northern states of India and has colder winters than the remaining sites and reports higher incidence of hyperreactive airway disease. Also, Chandigarh site was a referral tertiary care hospital with larger patient load.

A recent systematic review has reported results of 4 trials, (2 multi-centric hospital based studies and 2 community based cluster trials) [19]. They reported non inferiority of Oral amoxicillin administered either in hospital or community for treatment of severe pneumonia as compared to standard treatment by injectable penicillin. The two community based cluster trials compared oral amoxicillin administered at home by community health workers to administration of cotrimoxazole in the community followed by standard of care of receiving parenteral therapy at hospital. The hospital based studies compared the use of oral amoxicillin for management of WHO defined severe pneumonia to either injectable penicillin or ampicillin for 48 h. None of these studies compared the use of oral amoxicillin administered for the first 48 h under hospital supervision to ambulatory management at home with oral amoxicillin. This is the only study that not only reports the overall response of severe pneumonia to oral amoxicillin whether administered at home or hospital but also provides the cost effectiveness of the treatment.

### Is oral Amoxicillin cost effective?

The total cost of treating severe pneumonia in hospital for the first 48 h was significantly more than being treated at home. There were no significant differences between the two groups in costs of outpatient visits, but significantly higher costs were observed in the hospital group for the costs of hospitalization and for non-medical costs, which includes expenditure of the caregiver on travel or meal costs when taking care of the child. This was despite the fact that cost of hospitalization was perhaps an underestimate of the true costs as only variable costs were measured, and, because the costs of treatment and hospitalization are subsidized at Indian government hospitals. These results thus suggest that it will be cost efficient to manage children with WHO defined severe pneumonia at home with oral amoxicillin.

This is an important finding because WHO has recommended that children with severe pneumonia must be hospitalised which increases the cost of treatment due to daily bed charge, cost of services of medical personnel, cost of medications, monitoring, lost wages, cost of food for caretakers etc. If the treatment at home for a subgroup of severe pneumonia patients without high risk features, is as effective as treating in the hospital then it will be cost effective to recommend treatment guidelines for management of severe pneumonia at home.

### Can these results be generalized?

Exclusion of children with additional risk factors such as measles, severe malnutrition, and those with radiological consolidation could apparently limit the external validity of the results. However, these were uncommon reasons for exclusion as most seriously ill children with presence of danger signs would be immediately admitted to hospital and would not undergo the screening process. Of the 6634 patients of severe pneumonia that presented to the 6 tertiary care hospitals, only 16.9 % were eligible to participate to receive oral amoxicillin. The most common reason for exclusion in this study at screening was refusal of consent to participate if the child were to be randomized to hospital group. This trend was also observed after the randomization to hospital group when a large number of children left against medical advice and were declared treatment failure by definition despite no clinical deterioration. The other reasons for exclusion were wheeze or lower chest indrawing responding to nebulization (11 %) or having received an antibiotic for longer than 48 h (4.7 %). Thus, since children with co-morbid conditions were not included in the trial these results are largely generalizable to patients with severe pneumonia who don’t have co-morbidities or danger signs.

### Limitations and strengths

Excluding patients with consolidation could also exclude those with bacterial pneumonia, while those children with fever, wheeze and infiltrates are more likely to be of nonbacterial causes or viral pneumonias.

Children with allergic bronchitis, asthma, bronchiolitis and viral pneumonia can also manifest with clinical signs of WHO defined severe pneumonia of cough with LCI. Excluding children whose LCI disappeared after nebulization with bronchodilators or those with a past history of wheeze or bronchodilators administration ensured that we minimize inclusion of children with allergic bronchitis or asthma. In developing countries, mixed viral and bacterial infections are not uncommon, and hence need to be treated with antibiotics. However, admitting these children as severe pneumonia will be an additional cost for the Government and for the patient’s family.

The limitation in recording the cost data was that it was based partially on recall and partially on documents. It was also difficult to collect the cost data from patients who left against medical advice and those who had treatment failure. The sample size calculation was based on the clinical outcomes and not for the economic analysis. This may have influenced the validity of the cost effectiveness analysis.

Lastly, multiplicity of end points or a composite of end points of treatment failure that includes many conditions such as clinical deterioration, hospitalization, development of co-morbid conditions, changing antibiotics etc. do provide statistical efficiency but at the risk of difficulties with interpretation. Similarly, including treatment failures due to leaving against medical advice, lost to follow up or voluntary withdrawal can overestimate treatment failures and may not indicate true clinical deterioration, again causing difficulties with interpretation. Therefore, we analyzed treatment failure on intention to treat as well as per protocol basis.

Finally, this is the only trial that has evaluated the cost effectiveness and efficacy of oral amoxicillin administered at the hospital for first 48 h as compared to home administered oral amoxicillin to complete a course of 7 day treatment of WHO defined severe pneumonia in children at tertiary care hospitals as it not only reports the overall response of severe pneumonia to oral amoxicillin whether administered at home or hospital but also provides the costs of treatment and the differences in cost in the hospitalized and home group. This is an important strength of the study and will help greatly to guide policy for the case management of severe pneumonia in the developing countries.

## Conclusions

The study results suggest that in selected children aged 3–59 months with chest indrawing pneumonia and without any of the study exclusion criteria, home based treatment of with oral amoxicillin is equivalent to 48 h of hospital administered oral amoxicillin followed by home based treatment. However, the results of this study should be generalized with due consideration of the fact that the selected participants had a limited spectrum of presentation which may not be true in the real life scenario.

This study also concludes that cost of treatment of severe pneumonia with oral amoxicillin in the hospital for initial 48 h followed by continuing it at home for 5 days is significantly more than the cost of treatment with oral amoxicillin at home for 7 days.

Hence, consistent with the recent WHO simplified guidelines, it will be cost effective to manage select stable children with only fast breathing and chest-indrawing or WHO defined chest indrawing pneumonia at home with oral amoxicillin.

## Additional files

Additional file 1:
**Standard operating procedures that were used to train the physicians in assessing clinical signs.** (PDF 240 kb) Additional file 2:
**Details of contributorship of the ISPOT study group.** (PDF 288 kb)

## Abbreviations

ANOVA: Analysis of variances
ITT: Intention to treat
LAMA: Left against medical advice
LCI: Lower chest indrawing
OLS: Ordinary Least Squares Regression
Rs: Rupees
WHO: World Health Organization
