# Supplementary material for: A randomized controlled trial of hospital versus home based therapy with oral amoxicillin for severe pneumonia in children aged 3 – 59 months: The IndiaCLEN Severe Pneumonia Oral Therapy (ISPOT) Study
Source: BMC Pediatr. 2015 Nov 17;15:186. doi: 10.1186/s12887-015-0510-9 (PMC4650851; doi:10.1186/s12887-015-0510-9)
Supplement: Additional file 1: — Standard operating procedures that were used to train the physicians in assessing clinical signs. (PDF 240 kb) [file 12887_2015_510_MOESM1_ESM.pdf]

# Definitions & Assessment

## I. Inclusion Criteria

**1. Age 3 – 59 months :**

It means child completed 90 days and not completed 59 months at the date of enrollment.

**How to measure age:** Details of date of birth and/or age in months. If the exact date of birth is unknown the investigator will request the month of birth by event calendar and will assign the $15^{th}$ of that month as birth date.

**2. Lower chest wall indrawing:** defined as the inward movement of the bony structure of the lower chest wall with inspiration, is a useful indicator of severe pneumonia. It is more specific than "intercostal indrawing," which concerns the soft tissue between $3^{rd}$ -$5^{th}$ ribs without involvement of the bony structure of the chest wall. Chest indrawing should only be considered present if it is consistently present in a calm child. Agitation, a blocked nose or breastfeeding can all cause temporary chest indrawing. Any chest indrawing, even if it is not severe, is an indicator of severe pneumonia in a child aged 2 months up to 5 years.

If you have not already lifted the child's shirt when looking for fast breathing, ask the mother to lift it now, before you look and listen for lower chest indrawing, Stridor and wheeze. Before looking for these signs, make sure you know when the child is breathing **IN** and when the child is breathing OUT.

Look for lower chest indrawing when the child is breathing **IN.** The child has lower chest indrawing if the lower chest wall goes ***IN*** when the child breathes **IN.** Chest indrawing occurs when the effort required to breath in is much greater than normal. In normal breathing, when the child breathes **IN,** the whole chest wall (UPPER and LOWER) and the abdomen move OUT. If only the soft tissue between the upper ribs or above the clavicle goes in when the child breathes in this is not lower chest indrawing.

Be especially careful when looking for chest indrawing in young infants. Mild chest indrawing is normal in young infants because their chest wall is soft. However, severe chest indrawing (very deep and easy to see) is a sign of pneumonia.

Sub costal indrawing is sometimes confused with an accentuation of the angle between the ribs and the stomach which occurs in infants who have abdominal or "Paradoxical" breathing. In this case, the angle between the chest and stomach is more pronounced because the stomach goes out but the ribs do not go in. Thus, both lower chest wall indrawing and abdominal breathing both cause the angle between the chest and abdomen to be accentuated. Such an accentuation alone, should not be used to define subcostal indrawing.

If there is any question about whether the child has chest indrawing, reposition the child and look again. If the child is bent at the waist, it is hard to judge the movement of the lower chest wall. Reposition the child so he is lying flat in the mother's lap**.** ***If the chest indrawing is still not clearly visible, or if there is inter-observer variation assume that the child does not have chest indrawing.***

Chest indrawing is only significant if it is present all the time and definitely visible. If you see it only when the child is upset or trying to feed, but not when resting peacefully, do not consider this as lower chest indrawing.

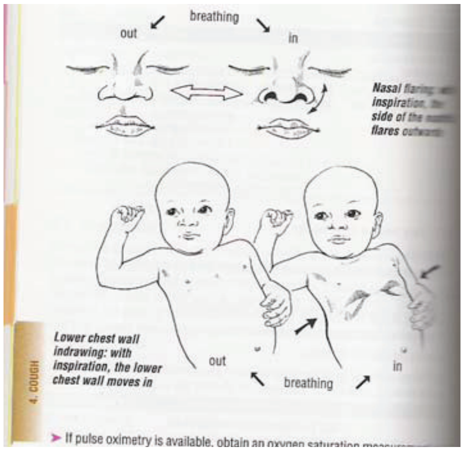


**3. Consent - Procedures for obtaining informed consent:**

The parents or legal guardians of the children eligible to enter the study will be fully informed about the study in his or her own first language. The study physician will obtain the freely given,

written consent of the parents or legal guardian for the child to participate in the study. The content of the explanation provided to the parents or legal guardians of the children is described in the attached consent form. If the parent or guardian is not literate, the right thumbprint may be substituted for signature, duly witnessed by somebody in addition to the person requesting consent. The information about the child will remain confidential.

## II Exclusion criteria

**4. Known or clinically recognizable chronic conditions :** Congenital cardiac or respiratory anomalies, chronic lung disease including broncho-pulmonary dysplasia, neurological impairment that affects respiratory function, renal diseases, malignant or hematological diseases.

**5. History of > 2 weeks of cough /difficulty in breathing:** Patient having H/O cough for >2 weeks or difficulty in breathing will be excluded from the trial

**6. Past history of more than 3 wheezing episodes or physician diagnosed asthma:** is to be ascertained by evidence/ documentation of bronchodilator (syrup or nebulization) in past 3 episodes. These patients may have respiratory distress without infection and most often do not need antibiotics. Children who had 3 or more episodes of wheezing in the past will also be excluded.

**7. LCI that responds to trial of nebulization:**

Children having had wheezing that improves after receiving bronchodilator therapy, as explained in the footnote1. These children may have asthma. Nebulization should not be driven by oxygen.

**8. RR ≥ 70 breaths per minute in calm child:**

**Respiratory Rate:** which distinguishes children who have pneumonia from those who do not; No single clinical sign has a better combination of sensitivity and specificity to detect

**1[Wheezing children will be given one dose of nebulized salbutamol/epinephrine (0.5 ml plus 2.0 ml of sterile water or 2 puffs using a metered-dose inhaler with a spacer device) and re-evaluated after 20 minutes. The only accepted sign of improvement is the absence of lower chest indrawing. For children still having LCI, a second dose of nebulization will be given and the child will be re-evaluated after another 20 minutes. In case of disappearance of LCI after receiving either dose of nebulization the child should not be included in the study. ]**

pneumonia in children under 5 than ***respiratory rate, specifically fast breathing***. Even auscultation by an expert is less sensitive as a single sign.

Cut-off rates for fast breathing (the point at which breathing is considered to be fast) depend on the child's age. Normal breathing rates are higher in children age 2 months up to 12 months than in children age 12 months up to 5 years.

**Child's Age Cut-off Rate for Fast Breathing**

2 months up to 12 months : 50 breaths per minute or more

12 months up to 5 years : 40 breaths per minute or more

**Note:** The specificity of respiratory rate for detecting pneumonia depends on the prevalence of bacterial pneumonia among the population. In areas with high levels of viral pneumonia, respiratory rate has relatively modest specificity. Nevertheless, even if the use of respiratory rate leads to some over treatment, this will still be small compared with the current use of antibiotics for all children with an ARI, as occurs in many clinics.

Using the timer, count the number of breaths taken in **one full minute**. Measure breathing rates only when the child is quiet, feeding or asleep.

Respiratory rate is to be measured twice. If the first two counts differ by more than 5, do a third count. Otherwise enter 999 for the third count.

**<u>How to use the Timer</u>**

- Push the Start/Stop button once. A short "beep" sound will be heard to show that the timer is starting. Actual counting of the respiratory rate must begin as soon as this initial beep is heard. Audible clicks will sound every second so that you can be sure that timing is in progress while you continue to observe the patient.
- The timer will give another short "beep" at the 30 second point of the timing cycle, and two short beeps at the end of 60 seconds when it will automatically shut off. The timer can be stopped at any time during the counting cycle, if needed, by pushing the Start/Stop button once.

## 9. Known HIV positive child or HIV status of mother known to be positive and of child not known/defined:

### *Clinical Categories For Children With HIV Infection*

**Category N: Not Symptomatic**

Children who have no signs or symptoms considered being the result of HIV infection or having only one of the conditions listed in Category A.

***Category A: Mildly Symptomatic***

Children with two or more of the conditions listed below but none of the conditions listed in Categories B and C.

- Lymphadenopathy (0.5 cm at more than two sites; bilateral = one site)
- Hepatomegaly
- Splenomegaly
- Dermatitis
- Parotitis
- Recurrent or persistent upper respiratory tract infection, sinusitis, or otitis media

*Category B: Moderately Symptomatic*

Children who have symptomatic conditions other than those listed for Category A or C, which are attributed to HIV infection. Examples of conditions in clinical Category B include but are not limited to the following:

- Anemia (<8 g/dL), neutropenia (<1000/mm 3 ), and/or thrombocytopenia (<100 000/mm 3 ) persisting for 30 d
- Bacterial meningitis, pneumonia, or sepsis (single episode)
- Oral Candidiasis or or pharyngeal (thrush), persisting (>2 months) in children >6 mo of age
- Cardiomyopathy
- Cytomegalovirus infection, with onset before 1 month of age
- Diarrhea, recurrent or chronic
- Hepatitis

- Herpes simplex virus (HSV) stomatitis, recurrent (>2 episodes within 1 year)
- HSV bronchitis, pneumonitis, or esophagitis with onset before 1 month of age
- Herpes zoster (shingles) at least 2 distinct episodes or involving more than 1 dermatome
- Leiomyosarcoma
- Lymphoid interstitial pneumonia (LIP) or pulmonary lymphoid hyperplasia complex
- Nephropathy
- Nocardiosis
- Persistent fever (lasting >1 month)
- Toxoplasmosis, onset before 1 month of age
- Varicella, disseminated (complicated chickenpox)

*Category C: Severely Symptomatic*

- Serious bacterial infections, multiple or recurrent (i.e., any combination of at least two culture-confirmed infections within a 2yr period), of the following types: septicemia, pneumonia, meningitis, bone or joint infection, or abscess of an internal organ or body cavity (excluding otitis media, superficial skin or mucosal abscesses, and indwelling catheter-related infections)
- Candidiasis, esophageal or pulmonary (bronchi, trachea, lungs)
- Coccidioidomycosis, disseminated (at site other than or in addition to lungs or cervical or hilar lymph nodes)
- Cryptococcosis, extra pulmonary
- Cryptosporidiosis or isospsoriasis with diarrhea persisting >1 month
- Cytomegalovirus disease with onset of symptoms at age >1 month (at a site other than liver, spleen, or lymph nodes)
- Encephalopathy (at least one of the following progressive findings present for at least 2 months in the absence of a concurrent illness other than HIV infection which could explain the findings): (1) failure to attain or loss of developmental milestones or loss of intellectual ability, verified by standard developmental scale or neuro-psychological tests; (2) impaired brain growth or acquired microcephaly demonstrated by head circumference measurements or brain atrophy demonstrated by computed tomography or magnetic resonance imaging (serial imaging is required for children <2 yr of age); (3) acquired symmetric motor deficit

manifested by 2yr or more of the following: paresis, pathologic reflexes, ataxia, or gait disturbance.

- Herpes simplex viral infection causing a mucocutaneous ulcer that persists for >1 month; or bronchitis, pneumonitis, or esophagitis for any duration affecting a child >1 month of age
- Histoplasmosis, disseminated (at a site other than or in addition to lungs or cervical or hilar lymph nodes)
- Kaposi's sarcoma
- Lymphoma, primary, in brain
- Lymphoma, small, non-cleaved cell (Burkitt's), or immunoblastic, or large-cell lymphoma of B-cell or unknown immunologic phenotype
- Mycobacterium tuberculosis, disseminated or extra pulmonary
- Mycobacterium, other species or unidentified species, disseminated (at a site other than or in addition to lungs, skin or cervical or hilar lymph nodes)
- Pneumocystis carinii pneumonie.
- Progressive multifocal leuco encéphalopathy.
- Toxoplasmosis of the brain with onset at >1 month of age
- Wasting syndrome in the absence of a concurrent illness other than HIV infection that could explain the following findings: (i) persistent weight loss >10% of base-line OR (ii) downward crossing of at least 2 of the following percentile lines on the weight-for-age chart (e.g., 95th, 75th, 50th, 25th, 5th) in a child 1yr of age OR (iii) <5th percentile on weight-for-height chart on 2 consecutive measurements, 30 days apart PLUS (i) chronic diarrhea (ie, at least 2 loose stools per day for 30days) OR (ii) documented fever (for 30 days, intermittent or constant)

**10. Hospitalization for >48 hours in the last two weeks:** This will exclude cases with possible nosocomial pneumonia that could require second line antibiotics

**11. Measles in the last month:** These patients may have immune suppression.

***Measles***. In measles, a red rash begins behind the ears and on the neck. It spreads to the face. During the next day, the rash spreads to the rest of the body, arms and legs. After 4 to 5 days, the rash starts to fade and the skin may peel. Some children with severe infection may have more

rash spread over more of the body. The rash becomes more discolored (dark brown or blackish), and there is more peeling of the skin. A measles rash does not have vesicles (blisters) or pustules. The rash does not itch. Do not confuse measles with other common childhood rashes such as chicken pox, scabies or heat rash. (The chicken pox rash is a generalized rash with vesicles. Scabies occurs on the hands, feet, ankles, elbows, buttocks and axilla. It also itches. Heat rash can be a generalized rash with small bumps and vesicles that itch. A child with heat rash is not sick).

Detection of acute (current) measles is based on fever with a generalized rash, plus at least one of the following signs: red eyes, runny nose, or cough. The mother should be asked about the occurrence of measles within the last month (recent measles). It is important to check every child with recent or current measles for possible mouth or eye complications. An infant with corneal clouding needs urgent treatment with vitamin A. Other possible complications such as pneumonia, stridor in a calm child, diarrhea, malnutrition and ear infection are assessed in relevant sections of these guidelines.

**12. Clinically Severe malnutrition:** defined as weight for length/height or clinical severe malnutrition( grade IV marasmus or kwashiorkor )

**Clinical Assessment**

Because reliable height boards are difficult to find in most outpatient health facilities, nutritional status should be assessed by looking and feeling for the following clinical signs:

***Visible severe wasting-*** This is defined as severe wasting of the shoulders, arms, buttocks, and legs, with ribs easily seen, and indicates presence of marasmus.

To look for visible severe wasting, remove the child's clothes. Look for severe wasting of the muscles of the shoulders, arms, buttocks and legs. Look to see if the outline of the child's ribs is easily seen. Look at the child's hips. They may look small when you compare them with the chest and abdomen. Look at the child from the side to see if the fat of the buttocks is missing. When wasting is extreme, there are many folds of skin on the buttocks and thigh. It looks as if

the child is wearing baggy pants. The face of a child with visible severe wasting may still look normal. The child's abdomen may be large or distended.

***Oedema of both feet -*** The presence of oedema (accumulation of fluid) in both feet may signal kwashiorkor. Children with oedema of both feet may have other diseases like nephrotic syndrome. There is a need, however, to differentiate these other conditions in the outpatient settings because referral is necessary in any case.

***Sunken eyes-*** The eyes of a dehydrated infant may look *sunken*. In a severely malnourished infant who is visibly wasted, the eyes may always look sunken, even if the infant is not dehydrated. Even though the sign "sunken eyes" is less reliable in a visibly wasted infant, it can still be used to classify the infant's dehydration.

***Elasticity of skin -*** Check elasticity of skin using the skin pinch test. When released, the skin pinch goes back either *very slowly* (longer than 2 seconds), or *slowly* (skin stays up for a brief instant), or *immediately*. In an infant with severe malnutrition, the skin may go back slowly even if the infant is not dehydrated. In an overweight infant, or an infant with oedema, the skin may go back immediately even if the infant is dehydrated.

After the infant is assessed for dehydration, the caretaker of an infant with diarrhea should be asked how long the infant has had diarrhea and if there is blood in the stool. This will allow identification of infants with persistent diarrhea and dysentery.

**13. Rickets: –** Palpable enlarged chostochondral junction (rachitic rosary. Thickening /widening of wrist and ankles

**14. Central Cyanosis: -** It could be peripheral, observed in the nail beds or central. Peripheral cyanosis can be present due to cold environment. Look for central cyanosis indicated by bluish discoloration in the mucous membrane of tongue and lips.

**15. Kerosene poisoning within last 48 hours:** Cough, increased RR after history of consuming kerosene in last 48 hours

**16. Oxygen saturation:** Pulse oximetry $< 80$ % on room air will be excluded.

**17. Abnormally sleepy or difficult to wake:**

An abnormally sleepy child is drowsy most of the time when the child should be awake and alert. This sick child will not look at the mother or watch your face when you talk. This child may stare blankly and may not appear to see. Ask the mother if the child has seemed unusually sleepy or difficult to wake. (Ask the mother if child was sleepy before also)

Look to see if the child wakens when the mother talks, or when you clap your hands. A child who is difficult to wake may continue to sleep even with the mother's voice or a loud clap. Even a very young baby, who sleeps a lot, should waken naturally with these disturbances, or when the mother begins to undress the child.

**Lethargy or unconsciousness.** Young infants often sleep most of the time, and this is not a sign of illness. Even when awake, a healthy young infant will usually not watch his mother and a physician/health worker while they talk, as an older infant or young child would. A lethargic young infant is not awake and alert when he should be. He may be drowsy and may not stay awake after a disturbance. If a young infant does not wake up during the assessment, flick the sole 2-3 times. Look to see if the child wakens and whether he stays awake. If the young infant shows no response or does not stay awake after some response, he is lethargic or unconscious.

**18. Inability to drink:** Do not rely completely on the mother's evidence for this, but observe while she tries to breastfeed or to give the child something to drink.

A child may be unable to drink either because s/he is too weak.

**19. Stridor in calm child:**

Look when the child is breathing **IN**. Stridor is a harsh noise made when the child breathes **IN**. Listen for Stridor by holding your ear near the child's mouth, since the noise may be difficult to hear. Stridor occurs when there is a narrowing of the larynx, trachea, or swelling of the epiglottis which interferes with air entering the lungs. These conditions are also called croup.

**20. Convulsions :** Convulsions may be associated with meningitis, cerebral malaria or other life-threatening conditions. On the other hand, convulsions may be the result of fever and in this instance, they do little harm beyond frightening the mother. All children who have had

convulsions during the present illness should be considered *seriously ill* because the more serious causes of convulsions cannot be differentiated from febrile convulsions without investigations conducted in a hospital.

**21. Known any antibiotic therapy for 48 hours or more immediately prior to admission**: Clinically, these children would be considered for a change of treatment to second line antibiotics on admission. Evidence of antibiotic use include any of the following: a) parental report that an antibiotics has been given; b) parent can provide evidence for prescription for antibiotics or c) has the container with antibiotic or d) recognizes medication when shown appropriate containers for locally available antibiotics. Children that have received antibiotics for less than 48 hours immediately prior to admission will be accepted in the trial.

**22. Other diseases requiring antibiotic therapy:** on presenting, such as meningitis, evident tuberculosis, dysentery, osteomyelitis, septic arthritis, etc. Use of other antibiotics needed for treatment.

**23.Persistent vomiting**: three or more episodes of vomiting within 1 hr

**24. Grunting** *:* Grunting is the soft, short sounds a young infant makes when breathing out. Grunting occurs when an infant is having difficulty in breathing.

**25. Known prior anaphylactic reaction to penicillin or amoxicillin:**

**26. Signs of severe dehydration according to WHO criteria:**

A young infant with **SEVERE DEHYDRATION** has any two of the following signs: is lethargic or unconscious, has sunken eyes, or a skin pinch goes back very slowly. Patients have severe dehydration if they have a fluid deficit equaling greater than 10 percent of their body weight. Young infants with severe dehydration require immediate IV infusion, nasogastric or oral fluid replacement according to WHO treatment guidelines described in Plan C (under treatment procedures).

**27. Severe pallor:**

**Clinical assessment**

**Palmer pallor:** Although this clinical sign is less specific than many other clinical signs included in the **IMNCI** guidelines, it can allow health care providers to identify sick children with severe anemia. The colour of the RO's palm can be compared with that of the child's palm to assess the paleness. Where feasible, the specificity of anemia diagnosis may be greatly increased by using a simple laboratory test for Hb estimation.

The most common cause of anemia in young children in developing countries is nutritional or because of parasitic or helminthes infections. However, there may be other more serious causes of anemia such as hemolytic anemia, aplastic anemia or leukemia.

**28. Suspected surgical pathology:** e.g.-1 Intestinal Obstruction

2 Hiatus Hernia

3 Inguinal Hernia

4 Intestinal Bands

5 Intestinal Polyp

6 Intussusceptions

**29. Living outside the catchments area (30 kms) of the study(30 kms):** These patients may be difficult to follow after leaving hospital.

**30. Subject previously included in the same trial or already included in another ongoing trials anywhere will be excluded.**

**31. Presence of radiological consolidation/effusion/pneumothorax:** Chest x ray will be obtained at each site and will use same definition for consolidation. CD of Digital radiographs of consolidation, effusion, pneumothorax will be sent to all the sites for standardization. (**Ref. WHO manual for Standardization of interpretation of chest radiographs for the diagnosis of pneumonia in children**)

**Note:** as soon as patient is back from radiology first rule out danger signs, SPO2< 80% and RR > 70/min. if any of these are present then patient is not included in the trial.
