# Supplementary material for: A randomized controlled trial of hospital versus home based therapy with oral amoxicillin for severe pneumonia in children aged 3 – 59 months: The IndiaCLEN Severe Pneumonia Oral Therapy (ISPOT) Study
Source: BMC Pediatr. 2015 Nov 17;15:186. doi: 10.1186/s12887-015-0510-9 (PMC4650851; doi:10.1186/s12887-015-0510-9)
Supplement: Additional file 2: — Details of contributorship of the ISPOT study group. (PDF 288 kb) [file 12887_2015_510_MOESM2_ESM.pdf]

Additional file 2. Details of contributorship of the ISPOT study group

Sponsors: IndiaCLEN and MCH-STAR

Concept, study design, and protocol development (alphabetical order)

Dr. Shally Awasthi
Dr. Luke Ravi Chellaiah
Dr. C.T. Deshmukh
Dr. LeAnne Fox
Dr. Tabish Hazir
Dr. Patricia Hibberd
Dr. S.K. Kabra
Dr. Sandhya Khadse
Dr. Ajith Krishnan
Dr. Rashmi Kumar
Dr. M.K.C. Nair
Dr. Archana Patel
Dr. Shamim Qazi
Dr. M.S. Rawat
Dr. Ashish Sabharwal
Dr. Sangeeta Saxena
Dr. Meenu Singh
Dr. Shruti Virmani

Monitoring of the study (alphabetical order)

Dr. Avinash Ansingkar
Dr. A.K. Patwari
Mr. Sameer Wadhwa

Data Safety & Monitoring Board (alphabetical order)

Dr. Piyush Gupta
Dr. Abhaya Indrayan
Dr. William Macleod (Chairperson)
Dr. Varinder Singh

Study Coordination

Dr. Archana Patel (PI)
Dr. Leena Dhande (CO-I)
Dr. Girish Charde (Study coordinator)
Ms. Smita Puppalwar (Program Manager)
Ms. Neetu Badhoniya (Statistician)

Development of study material & Data Management System

Dr. Archana Patel
Dr. Leena Dhande
Dr. Savita Bhargav
Dr. Dhanashree Uplap
M/s Expert Logic

Study implementation and data collection

Site	**Jawaharlal Nehru Medical College, Aligarh Muslim University, Aligarh**	**Post Graduate Institute of Medical Sciences, Chandigarh**	**Institute of Child Health, Chennai**	**Indira Gandhi Govt. Medical College & Lata Medical Research Foundation, Nagpur**	**B.J. Medical College, Pune**	**Mahatma Gandhi Institute of Medical Sciences, Sewagram**
Site Principal Investigator	Dr. Ashraf Malik	Dr. Meenu Singh	Dr. Luke Ravi Chellaiah	Dr. Archana Patel	Dr. Sandhya Khadse	Dr. Akash Bang
Co-Investigators	Dr. Uzma Firdaus<br>Dr. Meher Rizvi	Dr. Sadbhawna Pandit<br>Dr. Pallab Ray	Dr. C. Ravichandran<br>Dr.Md. Meeran	Dr. Leena Dhande<br>Dr. Gopal Agrawal	Dr. Chhaya Valvi	Dr. Manish Jain,<br>Dr. K Y Vilhekar,<br>Dr. D K Mendiratta,<br>Dr. Vijayshree Khairkar
Study Coordinator		Mr. Amit Agarwal	Dr. Saradha Suresh	Dr. Girish Charde		
Data collectors / Research officers	Dr. Mukhtar Ahmad<br>Dr. Shahbaz Hussain<br>Dr. Abu Anas<br>Mr. Mujahid Hassan (Social worker)	Dr. Rashmiranjan Das,<br>Dr. Nishant Jaiswal,<br>Dr. Abhishek,<br>Dr.Rajesh Kumar, Dr .Sukeshini,<br>Dr. Punish,<br>Dr. Sriniwas,<br>Dr. Muk mohit,<br>Dr. Monica<br>Devinder kumar (Social worker),<br>Harshkinder (Social worker),<br>Mr. Kundanlal (Technician),<br>Mr. S.D. Rana &<br>Mr. Nirmal Thakur (support staff)	Dr. R. Kannababu,<br>Dr. R. Thyagarajan<br>Dr. Jaikrishna<br>Dr. C.D. Indira Devi<br>Ms. J. Sakthivel (Social worker)<br>Mr. D. Rajesh (Social worker),<br>Parwathi (Support staff)	Dr. Savita Bhargava,<br>Dr.Dhanashree Uplap<br>Dr. Pravin Pachpande,<br>Dr.H. Markand,<br>Dr. Priya Bawiskar,<br>Dr.Sadaf Samreen,<br>Dr. Mohsin Akhtar<br>Dr.S.Tembhurne,<br>Dr.Pratibha Damle,<br>Dr. Nilesh Bhadke, Mr. N. Meshram (Social worker),<br>Mr. P. Chowdhary (Social worker), Mrs. Nalini Reddy (Technician)<br>Mr. Divesh Gadhia & Nilesh Thakre (support staff)	Dr. Parag Gadkari,<br>Dr. Sandeep Gajbhiye,<br>Ms. Savita Gawde,<br>Ms. Shaswati Patil	Dr. Suvarna Umarkar<br>Dr. Kiran Ingol<br>Dr.Bhushan Mhaiskar<br>Mr. Sanjay Jarunde (Social worker),<br>Mr. Manoj Chaudhary (Social worker)

Data Analysis (order of contribution)

Dr. Archana Patel
Ms. Neetu Badhoniya
Mr. Jitesh Borkar
Mr. P. Kumar Naidu

Data management &statistical analysis (order of contribution)

Dr. Archana Patel
Dr. Savita Bhargav
Ms. Neetu Badhoniya
Mr. Jitesh Borkar
Mr. P. Kumar Naidu

Report preparation (order of contribution)

Dr. Archana Patel
Ms. Sarah Hurlburt
Dr. Leena Dhande
Dr. Akash Bang
Dr. Meenu Singh
Dr. Saradha Suresh
Dr. Luke Ravi Chelliah
Dr. Uzma Firdaus
Mr. John M. Pile
Dr. Avinash Ansingkar
